# Differentiating combined pulmonary fibrosis and emphysema from pure emphysema: utility of late gadolinium-enhanced MRI

**DOI:** 10.1186/s41747-020-00187-w

**Published:** 2020-11-03

**Authors:** Hannah Fleming, Simon M. Clifford, Aoife Haughey, Roisin MacDermott, Niall McVeigh, Gerard M. Healy, Lisa Lavelle, Suhny Abbara, David J. Murphy, Aurelie Fabre, Edward McKone, Cormac McCarthy, Marcus Butler, Peter Doran, David A. Lynch, Michael P. Keane, Jonathan D. Dodd

**Affiliations:** 1 Department of Radiology, St. Vincent’s University Hospital, Elm Park, Dublin 4, Ireland; 2 School of Medicine, University College Dublin, Dublin, Ireland; 3 Department of Radiology, UT Southwestern Hospital, Dallas, TX USA; 4 Department of Pathology, St. Vincent’s University Hospital, Dublin, Ireland; 5 Department of Respiratory Medicine, St. Vincent’s University Hospital, Dublin, Ireland; 6 UCD Clinical Research Center, University College Dublin, Dublin, Ireland; 7 Department of Radiology, National Jewish Medical and Research Center, Denver, CO USA

**Keywords:** Bronchiectasis, Magnetic resonance imaging, Pulmonary emphysema, Pulmonary fibrosis, Tomography (x-ray computed)

## Abstract

**Background:**

Differentiating combined pulmonary fibrosis with emphysema (CPFE) from pure emphysema can be challenging on high-resolution computed tomography (HRCT). This has antifibrotic therapy implications.

**Methods:**

Twenty patients with suspected CPFE underwent late gadolinium-enhanced (LGE) thoracic magnetic resonance imaging (LGE-MRI) and HRCT. Data from twelve healthy control subjects from a previous study who underwent thoracic LGE-MRI were included for comparison. Quantitative LGE signal intensity (SI) was retrospectively compared in regions of fibrosis and emphysema in CPFE patients to similar lung regions in controls. Qualitative comparisons for the presence/extent of reticulation, honeycombing, and traction bronchiectasis between LGE-MRI and HRCT were assessed by two readers in consensus.

**Results:**

There were significant quantitative differences in fibrosis SI compared to emphysema SI in CPFE patients (25.8, IQR 18.4–31.0 *versus* 5.3, IQR 5.0–8.1, *p* < 0.001). Significant differences were found between LGE-MRI and HRCT in the extent of reticulation (12.5, IQR 5.0–20.0 *versus* 25.0, IQR 15.0–26.3, *p =* 0.038) and honeycombing (5.0, IQR 0.0–10.0 *versus* 20.0, IQR 10.6–20.0, *p =* 0.001) but not traction bronchiectasis (10.0, IQR 5–15 *versus* 15.0, IQR 5–15, *p* = 0.878). Receiver operator curve analysis of fibrosis SI compared to similarly located regions in control subjects showed an area under the curve of 0.82 (*p* = 0.002). A SI cutoff of 19 yielded a sensitivity of 75% and specificity of 86% in differentiating fibrosis from similarly located regions in control subjects.

**Conclusion:**

LGE-MRI can differentiate CPFE from pure emphysema and may be a useful adjunct test to HRCT in patients with suspected CPFE.

## Key points

- Late gadolinium-enhanced magnetic resonance imaging (LGE-MRI) can differentiate combined pulmonary fibrosis and emphysema from pure emphysema.
- LGE-MRI is not as accurate as high-resolution computed tomography (HRCT) in characterising reticulation or honeycombing.
- LGE-MRI may be a useful adjunct test to HRCT in differentiating combined pulmonary fibrosis and emphysema from pure emphysema.

## Background

Patients with combined pulmonary fibrosis and emphysema (CPFE) are characterised by a combination of fibrosis typically in the lower lobes and emphysema typically in the upper lobes [1, 2]. Identifying fibrosis in emphysema patients carries important prognostic and treatment implications and has been associated with an increased risk of pulmonary hypertension, progressive emphysema [3], and a higher all-cause mortality than either emphysema or fibrosis alone [4]. In the era of antifibrotic therapy, it has become even more relevant to differentiate those patients with combined fibrosis and emphysema from those with pure emphysema [5–7].

High-resolution CT (HRCT) is the most widely used imaging modality to diagnose CPFE [8]. A combination of reticulation and honeycombing are the principal fibrotic findings on HRCT. However, accurately identifying regions of definite fibrosis on HRCT can sometimes be challenging. Mimickers of honeycombing such as paraseptal emphysema may cause difficulty in confidently and accurately diagnosing cystic lung disease [9]. This is also true of cases with alternative fibrotic patterns to usual interstitial pneumonia [10]. An added issue is that spirometry may be difficult to interpret in CPFE, often showing appearances mimicking improvement in airflow obstruction because of progressive decreases in forced vital capacity over time [11]. Other patients may present with clinically suspected interstitial pulmonary fibrosis (IPF) but with obstructive spirometry. In both instances, accurate imaging of the underlying disease processes is important for diagnosis and treatment.

Cardiac MRI has become the reference-standard imaging test for detecting myocardial fibrosis [12]. A specific late gadolinium-enhanced (LGE) electrocardiographically gated segmented inversion recovery sequence allows the accurate depiction of fibrosis within the myocardium [13, 14]. The sequence relies on the concentration of the gadolinium-based contrast agent within the enlarged extracellular space of fibrotic tissue relative to normal myocardium.

It has been previously shown that pulmonary fibrosis can also be detected using this LGE-MRI in patients with IPF [15]. A key feature of this method, similar to myocardial fibrosis, is that it is the histopathological characteristics of fibrotic tissue, rather than its anatomical features, that allow the depiction of the fibrosis. In this study, we hypothesised that in patients with suspected CPFE, LGE-MRI would demonstrate contrast enhancement in regions of pulmonary fibrosis and no enhancement in regions of emphysema, allowing the differentiation of fibrosis from emphysema.

## Methods

**Fig. 1:**
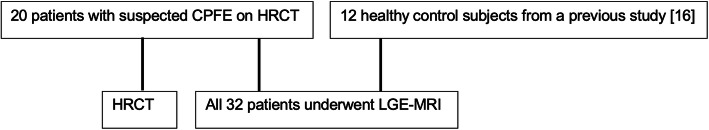
Flowchart of patients and controls. CPFE, Combined pulmonary fibrosis emphysema; HRCT, High-resolution computed tomography; LGE-MRI, Late gadolinium-enhanced magnetic resonance imaging

In this study, we retrospectively took into consideration the LGE-MRI scans acquired between April 2016 and March 2019 in 20 patients with CPFE suspected at HRCT. Patients were selected if there was difficulty in differentiating fibrosis from emphysema on HRCT in a multidisciplinary conference setting. This typically arose where lower lobe cysts were subpleural in location, had perceptible walls, and were round and clustered, features that are frequently seen in both fibrosis and emphysema [9]. Exclusion criteria included an acute chest infection or acute exacerbation of lung disease at the time of the investigations, contraindications to gadolinium-based contrast agents such as renal failure, and contraindications to MRI such as severe claustrophobia. The hospital Ethics Board approved the study and written informed consent was waived for this retrospective study. Data from twelve healthy control subjects from a previous study [15] who underwent LGE-MRI were included in the analysis for comparison. Body mass index was recorded for all subjects. Spirometry results were recorded when available. A flow chart diagram is provided in Fig. 1.

### High-resolution CT protocol

HRCT scans were acquired on a Siemens Sensation 64-slice CT (Siemens, Erlangen, Germany) from the apices to the costophrenic angles at full inspiration at 120 kVp and 130 mAs. All patients were scanned in the supine position. Contiguous slices were reconstructed at 1-mm slice thickness with a 0.5-mm increment and a 512 × 512 matrix. Automatic tube current modulation was utilised for all patients. Images were reconstructed using lung windows (window width 1,500, centre -700). Images were transferred to a workstation and analysed using quantitative lung analysis software (Pulmo3D, Syngo Via, Siemens, Erlangen, Germany) for calculation of total lung capacity, mean lung density, and the total low attenuation value % (LAV%) of the lungs. For the LAV% an established threshold of -950 HU was used to indicate emphysema [16].

### Thoracic MRI protocol

MRI scans were acquired on a 1.5-T magnet (Signa 1.5 T HDX, General Electric Healthcare, Milwaukee, Wis, USA) with an eight-element phased-array cardiac coil (General Electric Healthcare, Aurora, Ohio, USA). After a localising set of axial two-dimensional steady-state-free-precession images were acquired, a bolus injection of 0.2 mmol/kg of gadoterate meglumine (Dotarem, Gd-DOTA, Guerbet, Paris, France) was given, followed by a saline chaser bolus of 20 mL. This was followed 10 min later by a set of axial three-dimensional electrocardiographically gated segmented inversion-recovery prepared fast gradient-echo pulse sequences [17]. The 10-min delay was adapted from previously well-validated cardiac MRI protocols assessing myocardial fibrosis [18, 19]. Sequence parameters were as follows: matrix, 224 × 128; sensitivity encoding factor, 2; inversion time, 130–260 ms (individually optimised to null pulmonary artery blood signal); flip angle, 10°; in-plane resolution, 1.5 × 1.5 mm^2^; no slice gap; section thickness 1.5 mm resulting in a craniocaudal volume covering 2.5 cm per acquisition. Median number of acquisitions to cover the lungs from apex to base was 7 (interquartile range [IQR] 6–11, depending on the height of the patient). Each acquisition required a 10–15-s breath-hold. In order to null the contrast signal within the pulmonary circulation, inversion times were chosen based on the inversion time to null the blood pool in the pulmonary artery. We assessed the pulmonary artery null-time and adjusted accordingly for each acquisition, adapting similar principles from previously validated cardiac MRI approaches [20]. For patients unable to adequately breath-hold, we used nasal prongs oxygen delivery and a reduction in phase encoding steps to reduce sequence acquisition time. Scan time was recorded from the start to the end of image acquisition. Any complications such as contrast allergy were recorded.

### Image interpretation

Two radiologists with 15 and 2 years of experience read all scans in consensus and in random order, blinded to all clinical data. The LGE-MRI images were read blinded to the HRCT images and *vice versa*. For LGE-MRI, the signal intensity (SI) of fibrosis was measured by placing regions of interest (ROI) in regions of contrast-enhancement in CPFE patients. The ROIs were sized to avoid adjacent non-contrast lung and pulmonary vascularity. A similar process was performed for the HRCT scans. The SI of similar regions of lung in control subjects was also recorded for comparison. The SI of regions of emphysema was measured by placing ROIs in the right and left upper lobes in CPFE patients. A similar process was performed for the HRCT scans. The SI of similar regions of lung in control subjects was also recorded for comparison. LGE was defined using a SI threshold of > 3 standard deviations above the median SI of the upper lobes of the control subjects. The standard deviation of image noise was measured in an ROI outside the body to assess the contrast to noise ratio. The contrast-to-noise ratio was calculated by (median SI of the lung region of elevated SI − median SI of the upper lobe lung region)/1.5 × (standard deviation of noise), as used by others [18]. The percentage SI within pulmonary fibrosis relative to emphysema was calculated as 100 × (median SI of high SI lung region − median SI of upper lobe lung region)/(median SI of upper lobe lung region, adapted from previous myocardial fibrosis techniques [18]. The scoring system by Salisbury et al. [21] was adapted to score features of pulmonary fibrosis. Briefly, readers semiquantitatively scored the presence and extent of (i) reticulation, (ii) honeycombing, and (iii) traction bronchiectasis as follows: 0 (0% involvement of the lung with the feature), 1 (1–10% involvement), 2 (11–20%), 3 (21–30%), 4 (31–40%), 5 (41–50%); 6 (51–60%), 7 (61–70%), 8 (71–80%), 9 (81–90%), and 10 (91–100%). The midpoint of each category was arbitrarily used to convert the semiquantitative (0–10) to quantitative (0–100%) scores. For each scan, the percent involvement of each category for the right and left lungs were averaged to obtain a total lung score, and the radiologists’ total lung scores were averaged to obtain total volume occupied by each feature (HRCT was the reference standard in this study). The Fleischner society nomenclature recommendations were used to define abnormalities [22]. Scan quality for CT and MRI was scored as excellent, good, poor, or uninterpretable.

### Statistical analysis

Categorical data were expressed as number and percentage and continuous data as median and IQR. Comparisons of nominal values were performed using the *χ*^2^ test, and for scale values, the independent *t* test was used. Receiver operator characteristic (ROC) analysis was used to analyse the SI difference between regions of pulmonary fibrosis in CPFE patients with similar regions in control subjects to differentiate regions of pulmonary fibrosis from regions of dependent atelectasis (a *p* value was generated comparing the calculated AUC in the study to a hypothetical chance level AUC [= 0.5]). Sensitivity, specificity, positive and negative predictive values of LGE-MRI in characterising reticulation, honeycombing, traction bronchiectasis, and any fibrosis were calculated using HRCT as the reference standard.

All analyses were carried out using SPSS statistical software (version 13, SPSS Inc., Chicago, IL). A *p* value < 0.05 was considered to indicate a statistically significant result.

## Results

### Baseline data

The median age of the control group was significantly lower than the CPFE group (51.5 years, IQR 41.8–62.8 *versus* 72.0 years, IQR 67.8-77.3, *p* < 0.001) (Table 1). Median body mass index showed no significant difference between patients and controls (25.4 kg/m^2^, IQR 22.4–31.3 *versus* 25.4 kg/m^2^, IQR 23.0–29.2, *p* = 0.635). Spirometry was available in 14 patients and showed a median forced expiratory volume in one second of 2.4 L/s (IQR 2.1–3.0) and forced vital capacity of 3.1 L (IQR 2.5–3.8), indicating a group of patients with moderate emphysema. This was corroborated on quantitative CT with a median total lung capacity of 5.0 L (IQR 4.2–5.5), median lung density of -791 (IQR − 825–757), and median LAV% of 5.3% (IQR 1.4–12.3).

**Table 1:** Clinical characteristics of the study population

Demographics	Controls	CPFE
Age (years)	51.5 (41.8–62.8)	72.0 (67.8–77.3)*
Sex	6 males	14 males
BMI (kg/m^2^)	25.4 (23.0–29.2)	25.4 (22.4–31.3)
FEV_1_ (L/s)	NA	2.4 (2.1–3.0)
FVC (L)	NA	3.1 (2.5–3.8)
TLC (L)	NA	5.0 (4.2–5.5)**
MLD (HU)	NA	-791 (-825–757)**
LAV (%)	NA	5.3 (1.4–12.3)**

Results presented as median (interquartile interval)

*BMI* Body mass index, *CPFE* Combined pulmonary fibrosis and emphysema, *FEV*_*1*_ Forced expiratory volume in one second; *FVC* Forced vital capacity, *LAV%* Low attenuation value percent; *MLD* Mean lung density, *NA* Not assessed, *TLC* Total lung capacity

*p < 0.001

**Derived from quantitative computed tomography

### Scan data

All subjects successfully completed all MRI scans. There were no complications. Median time between LGE-MRI and HRCT was 58 days (IQR 21.8–140.0). There was a significant difference in scan time between controls and patients (26.0, IQR 23.3–30.0 *versus* 29.5, IQR 26.8–35.3, *p* = 0.041) related to hyperinflated lungs in the CPFE patients requiring increased scan time. MRI image quality was scored as excellent in 14, good in 13, fair in 4, and poor in 1 subject. CT image quality was scored as excellent in all cases.

### Control subjects

In control subjects, no significant differences were found in median SI between the upper and lower lobes (7.8, IQR 7.4-8.8 *versus* 6.3, IQR 0-18.1, *p* = 0.605) (Table 2). The percentage difference in SI in the lower compared to the upper lobes was -19.2%. The contrast-to-noise ratio was 2.3. Because images were blindly scored, a small number of control subjects were incorrectly scored as having pulmonary fibrosis, because of LGE in regions of dependent atelectasis. However, scores were very limited in extent, with median reticulation, honeycombing, and traction bronchiectasis extent scores of 0.5% (IQR 0.0–1.0), 0.0% (IQR 0.0–0.0), and 0.0% (IQR 0–1), respectively.

**Table 2:** Comparison of signal intensity between emphysema and fibrosis in CPFE patients and equivalently located regions in controls

	Controls	CPFE	*p* value
Emphysema	7.8 (7.4–8.8)	5.3 (5.0–8.1)	0.034
Fibrosis	6.3 (0.0–18.1)	25.8 (18.4–31.0)	0.001

Results presented as median (interquartile range)

*CPFE* Combined pulmonary fibrosis and emphysema

### CPFE patients

**Fig. 2:**
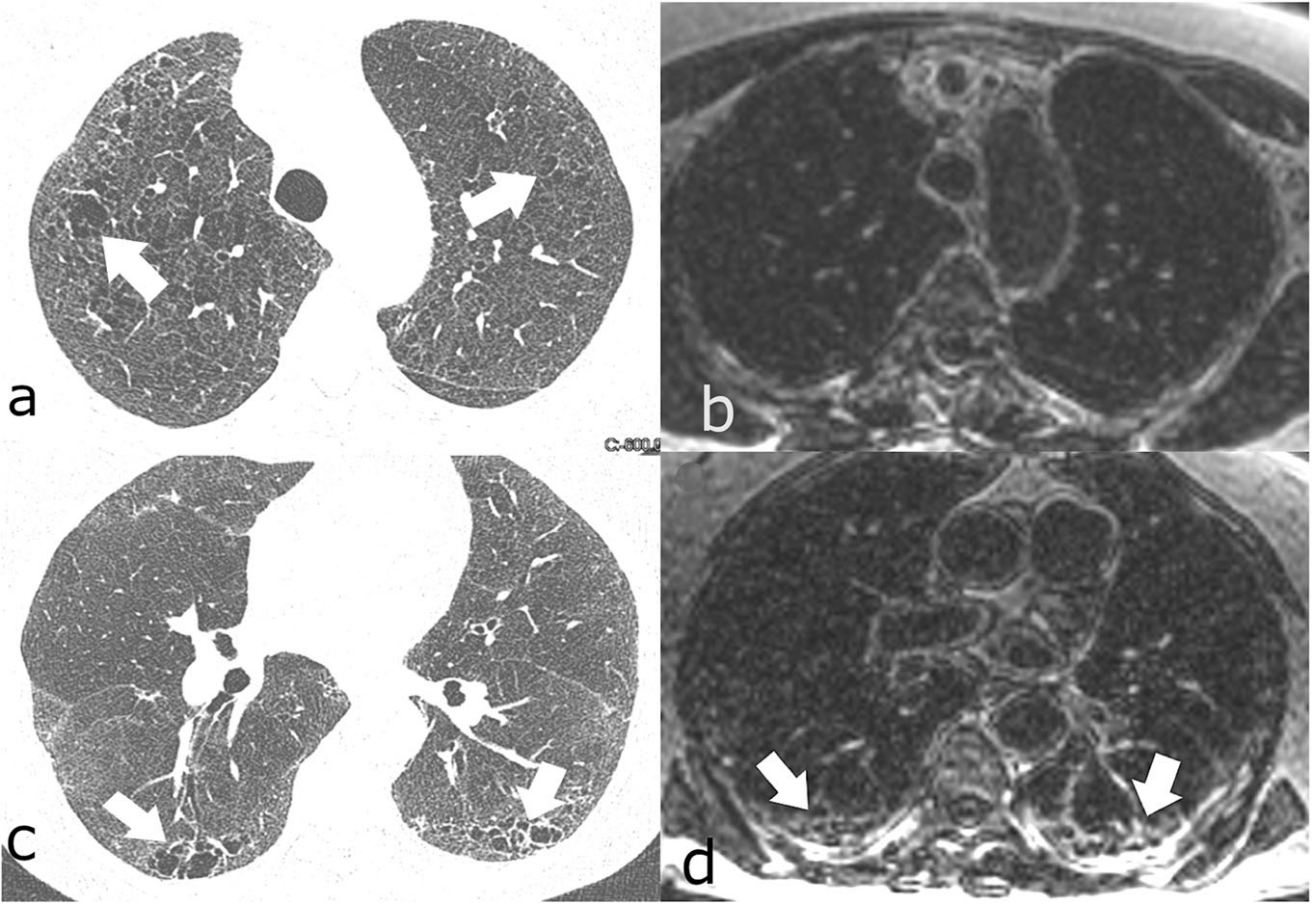
Seventy-two-year-old woman with progressive breathlessness, FEV_1_ 2.4 L/s, LAV 1.5%. **a** Axial high-resolution computed tomography showed mild centrilobular emphysema in the upper lobes (arrows). **b** It was unclear at multidisciplinary conference whether the abnormal regions in the lower lobes (arrows) represented emphysema or fibrosis. **c** Late gadolinium-enhanced magnetic resonance imaging of the upper lobes showed no contrast enhancement, but in **d**, definite contrast enhancement was observed in the abnormal regions in the lower lobes (arrows) and was consistent with fibrosis. *FEV*_*1*_, Forced expiratory volume in one second; *LAV%*, Low attenuation value percent

**Fig. 3:**
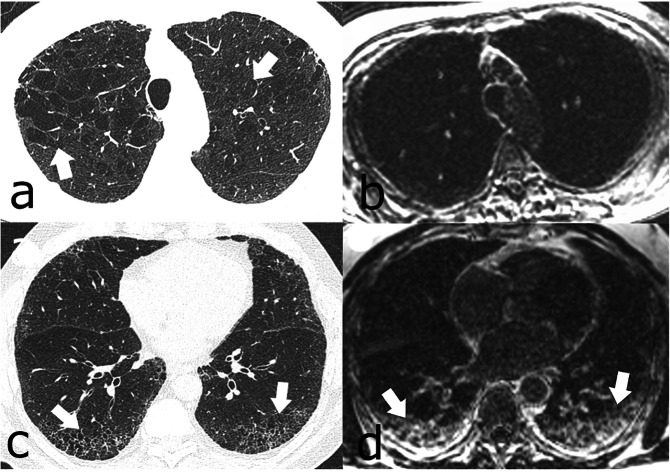
Fifty-seven-year-old man with progressive breathlessness, FEV_1_ 2.4 L/s, LAV 7.6%. **a** Axial high-resolution computed tomography showed moderate centrilobular emphysema in the upper lobes (arrows). **b** It was unclear at multidisciplinary conference whether the abnormal regions in the lower lobes (arrows) represented emphysema or fibrosis. **c** Late gadolinium-enhanced magnetic resonance imaging of the upper lobes showed no contrast enhancement, but in **d**, definite contrast enhancement in the abnormal regions in the lower lobes (arrows) was consistent with fibrosis. *FEV*_*1*_, Forced expiratory volume in one second; *LAV%*, Low attenuation value percent

**Fig. 4:**
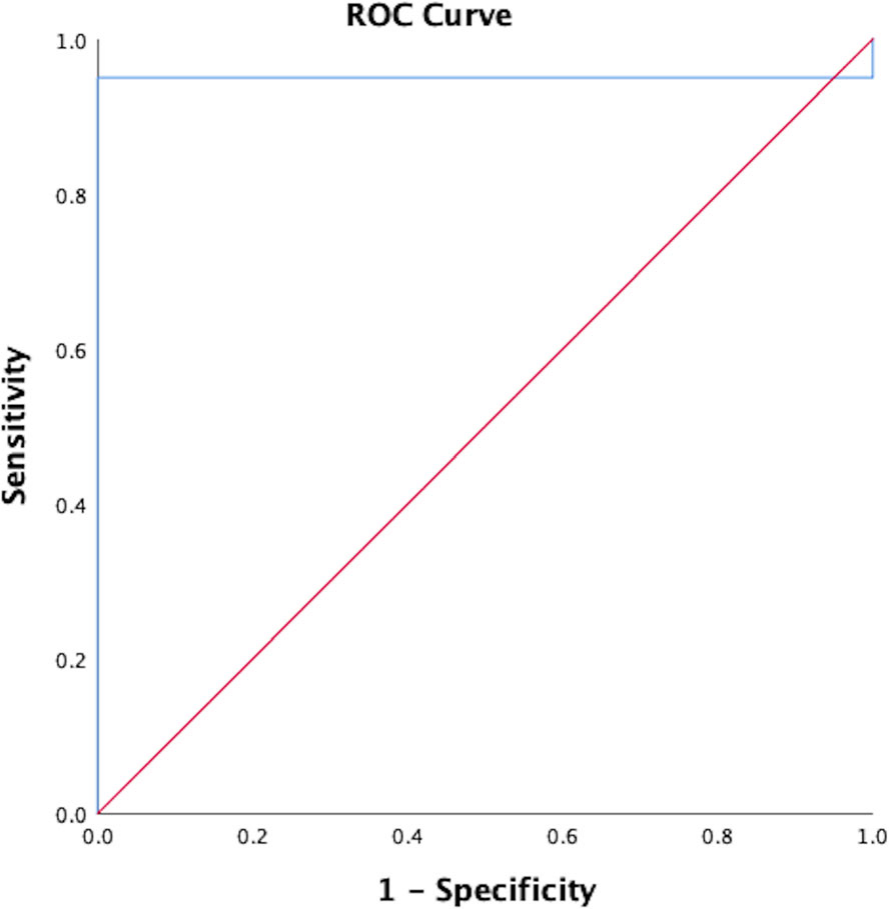
Receiver operator characteristic analysis of signal intensity in regions of fibrosis in the lower lobes compared to emphysematous areas on the upper lobes in CPFE patients. The area under the curve was 0.95, *p* < 0.001. Using a SI value of ≥ 12 resulted in a sensitivity of 95% and a specificity of 100% in differentiating fibrosis from emphysema in CPFE. *CPFE*, Combined pulmonary fibrosis and emphysema

All patients with CPFE were subjectively correctly identified on LGE-MRI. Figures 2 and 3 provide imaging examples in two patients with CPFE of LGE in regions of pulmonary fibrosis in the lower lobes and an absence of LGE in regions of emphysema in the upper lobes. The contrast-to-noise ratio was 30.1. Table 2 shows the median SI in regions of fibrosis and emphysema. Qualitatively, emphysema showed no evidence of LGE-MRI enhancement in any patient. Quantitatively, significant differences were found in median SI in regions of fibrosis compared to regions of emphysema (25.8, IQR 18.4–31.0 *versus* 5.3, IQR 5.0–8.1, *p* < 0.001). ROC analysis of SI in fibrotic areas in the lower lobes compared to emphysematous areas in the upper lobes in CPFE patients showed an area under the curve of 0.95, *p* < 0.0001. Using a SI value of ≥ 12 resulted in a sensitivity of 95% and specificity of 100% in differentiating fibrosis from emphysema in CPFE patients (Fig. 4). The percentage of SI increase in pulmonary fibrosis compared to emphysema was 278.5% while the percentage of density increase in pulmonary fibrosis compared to emphysema on HRCT was 36.4%. Significant differences were seen between LGE-MRI and HRCT in the extent of reticulation (12.5, IQR 5.0–20.0 *versus* 15.0–26.3, IQR 5–40, *p =* 0.038) and honeycombing (5.0, IQR 0.0–10.0 *versus* 20.0, IQR 10.6–20.0, *p =* 0.001) but not in traction bronchiectasis (10.0, IQR 5–15 *versus* 15.0, IQR 5.0–15, *p* = 0.878 (Table 3).

**Table 3:** Comparison of extent of pulmonary abnormalities on LGE-MRI and HRCT in CPFE patients

Abnormality	LGE-MRI	HRCT	*p* value
Reticulation	12.5 (5.0–20.0)	25.0 (15.0–26.3)	0.038
Honeycombing	5.0 (0.0–10.0)	20.0 (10.6–20.0)	0.001
Traction bronchiectasis	10.0 (5.0–15.0)	15.0 (5.0–15.0)	0.878

Results expressed as median (interquartile range). Extent of abnormalities is expressed as a percentage of total lung

*CPFE* Combined pulmonary fibrosis emphysema, *HRCT* High-resolution computed tomography, *LGE-MRI* Late gadolinium-enhanced magnetic resonance imaging

### Comparison between groups

**Fig. 5:**
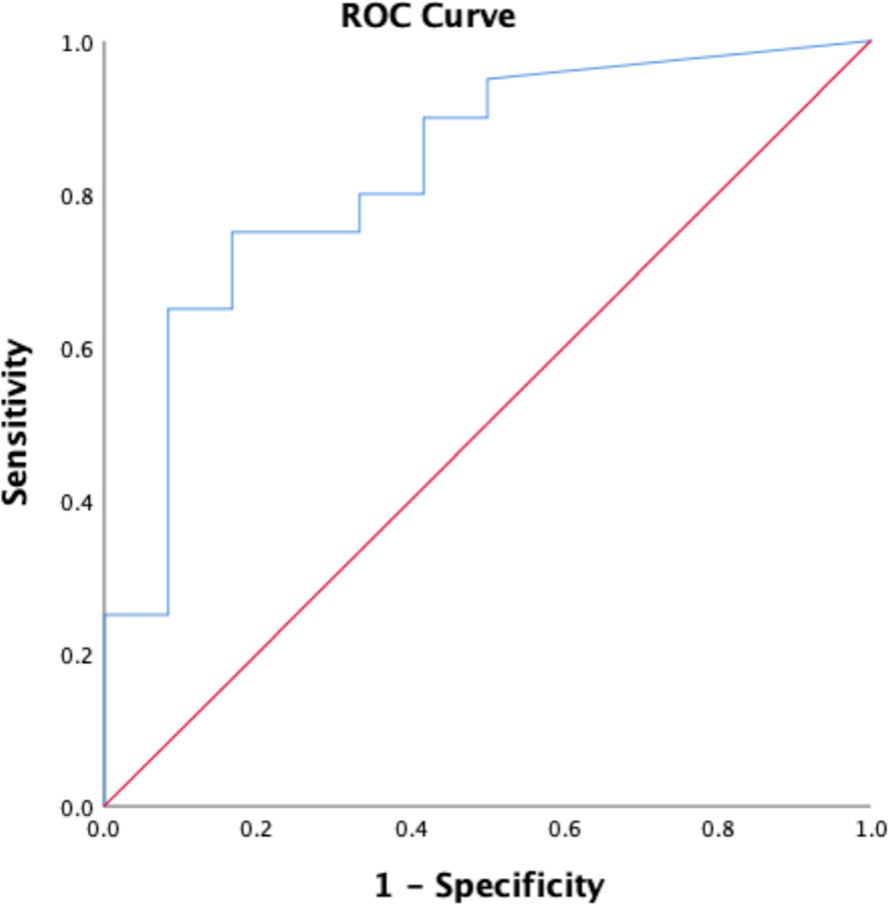
Receiver operator curve of signal intensity in regions of fibrosis in CPFE patients compared to similar regions of the lungs in control subjects. The area under the curve was 0.82, *p* = 0.002. Using a SI value of ≥ 19 resulted in a sensitivity of 75% and a specificity of 86% in differentiating fibrosis in CPFE patients from dependent atelectasis in controls. *CPFE*, Combined pulmonary fibrosis and emphysema

Significant differences were found in Si in regions of fibrosis in CPFE patients compared to equivalent lower lobe regions in control subjects (25.8, IQR 18.4–31.0 *versus* 6.3, IQR 0.0–18.1, *p* = 0.001). There was a significantly lower SI in regions of emphysema in CPFE patients compared to equivalent upper lobe regions in control subjects (5.3, IQR 5.0–8.1 *versus* 7.8, IQR 7.4–8.8, *p* = 0.034). ROC analysis of SI in regions of fibrosis in patients suspected of CPFE compared to similarly located regions in control subjects (typically in dependent regions of the lower lobes) showed an area under the curve of 0.82, *p* = 0.002 (Fig. 5). Using a signal cutoff intensity value of ≥ 19 resulted in a sensitivity of 75% and specificity of 86% in differentiating regions of fibrosis from similarly located regions in control subjects. A *post hoc* power sample size calculation for the ROC analysis showed that for an area under the curve of 0.82 for a cohort with 20 positive and 10 negative cases and an alpha error of 0.05 yielded a power of 90%. Table 4 shows that in cases of LGE-MRI where SI was ≥ 19, LGE-MRI had a high sensitivity and specificity in determining reticulation, traction bronchiectasis, and any fibrosis but a lower sensitivity (65%) in determining honeycombing.

**Table 4:** Sensitivity, specificity, and predictive values of LGE-MRI in determining the presence of pulmonary abnormalities on HRCT in CPFE patients

Abnormality	Sensitivity	Specificity	PPV	NPV
Reticulation	0.96	0.82	0.93	0.90
Honeycombing	0.65	0.92	0.93	0.63
Traction bronchiectasis	1.00	0.92	0.95	1.00
Any fibrosis	1.00	0.85	0.91	1.00

*CPFE* Combined pulmonary fibrosis emphysema, *HRCT* High-resolution computed tomography, *NPV* Negative predictive value, *PPV* Positive predictive value

## Discussion

The main finding from our study is that LGE-MRI may allow the differentiation of CPFE from pure emphysema alone. Its high sensitivity and specificity supports the concept that in patients where differentiating fibrosis from emphysema is challenging on HRCT, LGE-MRI may be a useful adjunct test. Although HRCT is the accepted reference standard for imaging interstitial lung fibrosis, its limitations in differentiating cystic lung disease are well published [9]. In our experience, differentiating pulmonary fibrosis from emphysema can be particularly challenging when there is an extensive paraseptal emphysema component. With several trials demonstrating the benefits of antifibrotic therapy in both IPF and non-IPF cohorts [6, 23], identifying patients with combined fibrosis and emphysema from pure emphysema is becoming increasingly relevant.

As shown in a previous work [24], the most critical aspect of the MRI sequence is identifying the correct inversion time to null the pulmonary artery blood signal. Nulling the signal from the pulmonary artery blood pool allows contrast detection in regions of fibrosis but minimises it in the pulmonary circulation [15]. This is why our scan times varied considerably between patients, because it takes time to identify the correct inversion time to null the blood signal from the pulmonary artery.

There are several differences when applying LGE-MRI to the lungs compared to the heart. Firstly, in cardiac imaging, LGE-MRI relies on nulling healthy non-fibrotic myocardial tissue [25]. Fibrotic myocardium becomes detectable because the null time for normal myocardium differs by about 200 ms from the null time of myocardial fibrosis [26]. In contrast, pulmonary fibrosis has an inversion time closer to the pulmonary blood pool, the difference being 40–60 ms. Thus, the increased SI in pulmonary fibrosis does not tend to be as high as it is in myocardial fibrosis. Nevertheless, there were significant qualitative and quantitative differences in the median percentage elevation in LGE-MRI within pulmonary fibrosis when compared to the SI of emphysema or control subjects. A much lower percentage increase in tissue density was obtained when similar measurements were repeated on HRCT.

To our knowledge, the application of LGE-MRI in patients suspected of CPFE has not been previously assessed. In a study of patients with IPF, LGE-MRI correctly identified all 20 IPF patients compared to controls [15]. In that study, median SI of the LGE within pulmonary fibrosis was 31.8 ± 10.6 *versus* 10.5 ± 1.6 for normal lung regions (*p* < 0.001), resulting in a percent SI elevation from pulmonary fibrosis of 204.8% ± 90.6. The results from the present study are similar although some controls were incorrectly scored as positive for fibrosis, related to regions of dependant atelectasis. We were unable to image patients in prone position to minimise this issue because of the requirement for vector electrocardiographic gating leads to be placed in specific anterior chest locations; placing the patient prone interfered with the electrocardiographic signal. Nevertheless, we found at ROC analysis that a SI cutoff of ≥ 19 allowed the differentiation of CPFE from areas of dependent atelectasis in controls with high sensitivity and specificity. We also found LGE-MRI was not as accurate as HRCT at characterising reticulation and honeycombing. As shown in Table 4, LGE-MRI had a lower specificity in differentiating reticulation from honeycombing. Despite the excellent spatial resolution of the three-dimensional LGE-MRI sequence, thin septal lines < 1 mm in thickness were at times difficult to identify, and we were unable to differentiate thin reticulation from honeycombing in some lung regions.

Several limitations of our study are noted. We do not have tissue pathological confirmation of lung fibrosis, as we followed current recommendations from the American Thoracic Society/European Respiratory Society guidelines for patients with CPFE [2, 27]. This is an issue because some lung regions that showed contrast enhancement on LGE-MRI may have represented true fibrosis, despite not being scored as fibrosis on the corresponding HRCT images. Nevertheless, we used HRCT because it is the current consensus imaging reference standard for pulmonary fibrosis [28].

It was not our intention to assess the optimum delay between contrast injection and MRI acquisition. We applied a 10-min delay because that is the standard protocol for depicting LGE-MRI in protocols for myocardial fibrosis [19]. For pulmonary fibrosis, it may be better to wait a longer time before acquisition, which might allow further washout of contrast from the pulmonary blood pool and help depict fibrosis more optimally. Further studies are needed to optimise these timing aspects. The LGE-MRI technique does require the administration of intravenous contrast, so is not suitable in all patients, for example those with severe renal failure. We did not assess inter-reader variability or reader experience in this study, which is an important aspect to evaluate in the future. We did not assess the technique across multiple vendors and this is another aspect to assess in future studies. Finally, while we have shown that LGE-MRI allows the quantitative analysis of pulmonary fibrosis and emphysema, imaging biomarkers for HRCT are undergoing rapid development [29]. Future studies comparing both modalities should provide further insights into which give more information in the quantitative analysis of CPFE.

In conclusion, LGE-MRI may differentiate CPFE from pure emphysema. It may be a useful adjunct test to HRCT in patients with suspected CPFE.

## Data Availability

The datasets used and/or analysed during this study are available from the corresponding author on reasonable request.

## Abbreviations

CPFE: Combined pulmonary fibrosis and emphysema
HRCT: High-resolution computed tomography
IPF: Interstitial pulmonary fibrosis
IQR: Interquartile range
LAV: Lung attenuation volume
LGE: Late gadolinium-enhanced
ROC: Receiver operating characteristic
ROI: Region of interest
SI: Signal intensity
